# Absence of Hofmeister Selectivity in Hydrophobic Ion-Exchanger Nanopores

**DOI:** 10.1021/acs.analchem.5c05239

**Published:** 2025-12-01

**Authors:** Gergely T. Solymosi, Tünde Kis, Péter Fürjes, Róbert E. Gyurcsányi

**Affiliations:** † BME Lendület Chemical Nanosensors Research Group, Department of Inorganic and Analytical Chemistry, Budapest University of Technology and Economics, Műegyetem Rkp. 3, Budapest H-1111, Hungary; ‡ Institute of Technical Physics and Materials Science, HUN-REN Centre for Energy Research, Konkoly Thege Miklós út, Budapest 29-33, H-1121, Hungary; § HUN-REN-BME Computation Driven Chemistry Research Group, Department of Inorganic and Analytical Chemistry, Budapest University of Technology and Economics, Műegyetem Rkp. 3, Budapest 1111, Hungary

## Abstract

Dehydration governs ion selectivity in both natural ion channels and synthetic ion sieves, as ions must dehydrate to traverse subnanometer pores. Because lipophilic ions experience lower dehydration energy costs, an intrinsic lipophilic selectivity pattern known as the Hofmeister series arises. This Hofmeister selectivity is modulated in natural ion channels by electrostatic and coordinative interactions between ions and nanopore surfaces, yielding diverse selectivities. Ion selectivity has also been demonstrated in larger synthetic nanopores—up to 5 nm in diameter—functionalized with ionophores that enhance specific coordinative interactions. In these synthetic ion channels, surface hydrophobicity is critical for ion selectivity; however, its exact role remains undetermined. Here, we elucidate the contribution of hydrophobicity by comparing structurally identical hydrophilic and hydrophobic cation-exchanger gold nanopores modified with 10-mercaptodecane-1-sulfonate, a ligand lacking strong ion-coordinating functionalities. Zero-current potentiometry revealed that cation-exchanger nanopores do not discriminate among singly charged cations, except for a modest preference for H^+^. Remarkably, this behavior was observed in both hydrophilic and nanometer-wide hydrophobic nanopores, contrasting with the strong Hofmeister selectivity of subnanometer pores and hydrophobic bulk polymer membranes. The absence of Hofmeister selectivity was confirmed in both multipore membranes and single nanopores, ruling out ensemble nonidealities as an explanation. These findings indicate that surface hydrophobicity alone does not impart lipophilicity-driven discrimination to cation-exchanger nanopores. The lack of intrinsic Hofmeister selectivity may enable unique ion-sensing applications (e.g., total ion concentration measurements, ligand-tuned ion-selective sensors) by minimizing interference from lipophilic species that commonly affect polymer-based sensing membranes, while still excluding bulk electrolyte from the pores.

## Graphical abstract

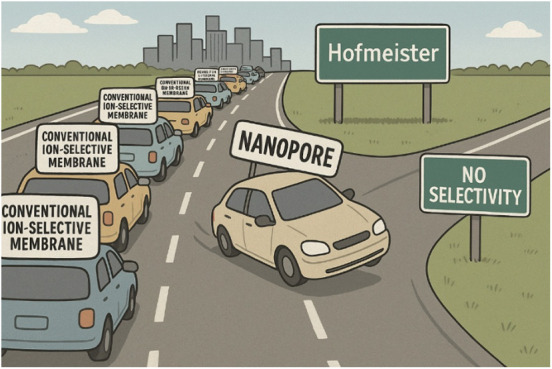

## Introduction

Nanopores have emerged as efficient and versatile tools for chemical analysis and separation, addressing a wide range of challenges from DNA^1^ and peptide sequencing^2^ to water desalination.^3,4^ These exceptional capabilities arise from their ability to differentiate and selectively transport ions and molecules based on size and physicochemical properties, such as charge or polarity. Nanopores achieve this due to their lumen being commensurate with both the sizes of ions and molecules and the range of various chemical and physical interactions, allowing them to function as molecular sieves or interaction-based filters.

Natural ion channels serve as exemplary models for designing highly selective nanopores. They can distinguish between ions with extremely high selectivity—up to 10^4^ even for closely similar species—by exploiting subtle differences in size and charge. This selectivity arises from subnanometer-scale filters that replace the ions’ hydration shells with precisely positioned coordinating functional groups on the channel walls.^5,6^ The selectivity of the ion channel is determined by the dehydration and coordination energies of the ions.^7^ The geometry and composition of the selectivity filter are precisely tailored to best accommodate the preferred ion, making its entry energetically favorable relative to other ions.^8,9^ Highly selective natural ion channels exist for several ions (including K^+^,^10^ Na^+^,^11^ Ca^2+^,^12^ Cl^–^,^13^ F^–^,^14^ Cu+^15^ ), which can be reproducibly produced using microorganisms and site-specifically modified via genetic engineering.^12,16^ However, their composition as lipid bilayer-embedded proteins limits their chemical and mechanical stability.^17^

The development of solid-state ion channels is thus critical to the advancement of next-generation nanopore technologies, especially in scalable, application-oriented settings.^17,18^ Solid-state nanopores can be fabricated by various methods (e.g., electron/ion beam milling,^19–21^ focused laser optical etching,^22^ controlled dielectric breakdown,^23^ laser pulling of capillaries,^24,25^ track-etching^26,27^ ) in diverse materials (e.g., silicon and aluminum oxide,^28^ silicon and boron nitride,^29^ graphene,^30^ graphene oxide,^4^ gold,^31^ various polymers^32^) with pore sizes down to a few Ångströms.^33^ These solid-state nanopores, however, lack the precisely located functional groups (e.g., carbonyls, carboxylic acids, and amines) that facilitate the diverse selectivities of natural ion channels.^33^ Subnanometer pores can still transport ions with high selectivities, operating as molecular sieves that impede the passage of ions with hydrated diameters larger than the pores by necessitating their dehydration.^4,34,35^ The selectivity of such ion sieves, however, are mostly limited to a hydration energy-dependent order (i.e., K^+^ > Na^+^ > Li^+^ > Ca^2+^ > Mg^2+^) known as the Hofmeister series, as more hydrophilic ions incur a higher cost for dehydration.^36^ In contrast, nanopores that can accommodate ions in a fully hydrated state do not discriminate based on hydration energy.^37–39^ For example, hydrophilic sulfonated nanopores with diameters >2 nm (i.e., significantly larger than hydrated ions) exhibit no preference for lipophilic ions.^40,41^ These charged, water-filled nanopores are selective for ions with smaller hydrodynamic radius^40^ and higher charge,^41^ attributed to the stronger electrostatic interaction with the sulfonated pore wall. However, the observed selectivities are minimal (<1 order of magnitude), indicating that electrostatic interactions alone in wide (i.e., > 2 nm) nanopores cannot discriminate efficiently between ions.

To achieve enhanced non-Hofmeister selectivities, more specific interactions between ions and the nanopore are required—such as the coordination of ions by carefully positioned functional groups on the pore surface. These interactions are significantly shorter-ranged than electrostatic forces, typically operating over only a few Ångströms.^42^ Therefore, achieving high selectivity necessitates confining ion transport to the immediate vicinity of the pore surface, as exemplified by the Ångström-scale selectivity filters found in natural ion channels. Replicating this behavior in solid-state systems remains challenging due to the difficulty of fabricating nanopores with subnanometer-level precision in materials that can also support such selective interactions. Alternatively, selective functionalities can be added to the solid-state nanopores postfabrication by the chemical modification of their surface.^33^ A wide range of selective binding molecules (e.g., metal chelating ligands,^43^ nucleic acids,^44–46^ peptides^47,48^ ) has been employed to turn solid-state nanopores into chemical sensors for various analytes.^49^ In most of these sensors, analyte binding modulates ion transport across the nanopore by altering either the effective pore cross-section or the surface charge.^49^ The functionalization of the nanopore, however, can also enable the selective transport of the analyte itself. Utilizing the latter approach, highly selective ionophore-based synthetic ion channels with diameters up to ∼5 nm have been developed for Ag^+^ and Cu^2+^ by rendering the walls of gold nanopores both selectively ion-coordinating and strongly hydrophobic through surface modification with mixtures of thiol-bearing ionophores and ion exchangers, and alkanethiols.^50,51^ The key to achieving high selectivity in nanometer-wide pores likely lies in the exclusion of bulk aqueous electrolytes from the nanopore lumen, as evidenced by the strong correlation between the hydrophobicity of the modified surface and the ion selectivity.^51^

Given the critical role of surface hydrophobicity in the selectivity of solid-state nanopores, here we aim to evaluate its effect by using hydrophilic and hydrophobic cation-exchanger nanopores that are otherwise identical. To eliminate confounding contributions from size-based ion sieving, we fabricated cylindrical gold nanopores (GNPs) by electroless gold plating of track-etched polycarbonate ultrafiltration membranes, yielding average pore diameters of 6 and 20 nm—dimensions significantly larger than those of hydrated ions.^44,52^ This approach produced multipore gold membranes with high pore density. Exploiting the well-established thiol–gold surface chemistry, which enables the formation of multicomponent self-assembled monolayers (SAMs), we prepared both hydrophilic and hydrophobic cation-exchanger GNP membranes. Specifically, 10-mercaptodecane-1-sulfonate (MDS) was employed to impart cation-exchange functionality, while a mixed MDS/1-decanethiol (DT) monolayer was used to introduce hydrophobicity. Sulfonate was selected as the ion exchanger to minimize ion coordination and the pH dependence of surface charge.

We assessed the ion selectivity of the cation-exchanger GNP membranes using zero-current potentiometry and benchmarked their performance to that of ion-exchanger plasticized PVC membranes, which are known to exhibit Hofmeister selectivity.^54^ To eliminate any averaging effect and extend our study to individual pores, hydrophilic and hydrophobic cation-exchanger *single* gold nanopores (single GNPs) of similar size were fabricated by focused ion beam (FIB) milling followed by the same thiol-based surface modifications as for the multipore membranes.

## Experimental Section

### Chemicals

All inorganic salts, acids, and bases used in this study were of analytical grade and procured from Merck (Germany) or Sigma-Aldrich (US). Aqueous solutions were prepared using ultrapure deionized water sourced from a Millipore Direct-Q 3 UV water purification system (Merck Millipore, US). Absolute ethanol of A.R. grade was acquired from Molar Chemicals (Hungary). Emplura grade methanol, Selectophore grade tetrahydrofuran (THF), ReagentPlus grade trifluoroacetic acid, 1-decanethiol (DT), and ACS reagent-grade methanol-stabilized 37% formaldehyde solution were obtained from Sigma-Aldrich (US). The Oromerse SO Part B gold plating solution was supplied by Technic Inc. (US). Selectophore-grade sodium tetrakis­[3,5-bis­(trifluoromethyl)­phenyl]­borate (NaTFPB), 2-nitrophenyl-octylether (o-NPOE), and high-molecular-weight poly­(vinyl chloride) (PVC) from Fluka (Switzerland) were employed in the preparation of ion-exchanger plasticized PVC membranes. Sodium 10-mercapto-1-decanesulfonate (NaMDS) was synthesized following the protocol of Turyan and Mandler for the functionalization of the GNP membranes.^55^

### Fabrication of Functionalized GNP Membranes

GNP membranes were made via electroless gold plating of track-etched filter membranes. Hydrophilic (polyvinylpyrrolidone-coated) polycarbonate filter membrane disks with a diameter of 25 mm, thickness of 6 μm, pore density of 6 × 10^8^ pore/cm,^2^ and nominal pore diameter of 30 nm were obtained from Cytiva (US). We used a modified version^44^ of the electroless gold plating protocol introduced by Martin’s group.^52^ The average inner diameter of the nanopores in the GNP membranes was determined by N_2_ gas permeation measurements.^51^ We optimized the plating times for preparing 6 and 20 nm mean pore diameter gold membranes. The detailed procedures are provided in the SI.

The GNP membranes were functionalized at 25 °C by overnight immersion into dilute, stirred ethanolic solutions of the thiol derivatives. Hydrophilic cation-exchanger membranes were functionalized with 0.1 mM NaMDS and hydrophobic cation-exchanger membranes with a mixture of 0.05 mM NaMDS and 0.05 mM DT (1:1 molar ratio). After functionalization, the GNP membranes were washed first with ethanol and then with deionized water. Finally, the membranes were dried in a vacuum.

### Fabrication of Functionalized Single GNPs

The single GNPs were fabricated by focused ion beam (FIB) milling in a submicron-thick multilayer membrane—consisting of a 200 nm-thick nonstoichiometric silicon nitride (SiN_*x*_) supporting layer, a 5 nm-thick titanium (Ti) adhesion layer, and a 150 nm-thick gold (Au) layer—that was suspended on a 380-μm-thick silicon (Si) frame (Figure S5). The membrane containing the single GNP and the Si frame are collectively referred to as the single GNP chip. The fabrication is detailed in the SI. The single GNPs had a conical shape with a half-cone angle of ca. 5° and the base of the cone on the SiN_*x*_ side of the SiNx/Au membrane. The smallest diameter of the conical pore was determined individually for each single GNP by scanning electron microscopy (SEM) immediately after fabrication using the same cross-beam setup of the Thermo Scientific Scios 2 DualBeam FIB-SEM. The single GNPs were functionalized in the same way as the multipore GNP membranes.

### Preparation of PVC-Based Cation-Exchanger Membranes

Cation-exchanger plasticized PVC membranes were made of 80 mg (66%) o-NPOE, 40 mg (33%) PVC, and 1 mg (0.8%) potassium tetrakis­[3,5-bis­(trifluoromethyl)­phenyl]­borate (NaTFPB). The materials were dissolved in 1 mL tetrahydrofuran (THF). The solution was homogenized first with a vortex mixer for 1 min and then with a tube roller mixer for 24 h. The homogenized solutions were cast in a 25 mm-diameter glass ring, the rings were covered with watch glass, and the THF was let to evaporate.

### Electrochemical Measurements of Single GNPs

Single GNP chips were sandwiched between two disk-shaped holders that contained outward-opening cone-shaped holes in their center to allow contact between the single GNPs and the electrolyte solutions. The holders were padded with Parafilm O-rings to ensure watertight sealing between the holders and the single GNP chip. The holder-chip assembly was mounted in a two-chamber Teflon transport cell (Figure 1B). The chambers were filled with aqueous electrolyte solutions, and double-junction Ag/AgCl/3 M KCl//1 M KCl//reference electrodes (Metrohm, Switzerland) were immersed into the solutions for electrical contact.

**Figure 1:**
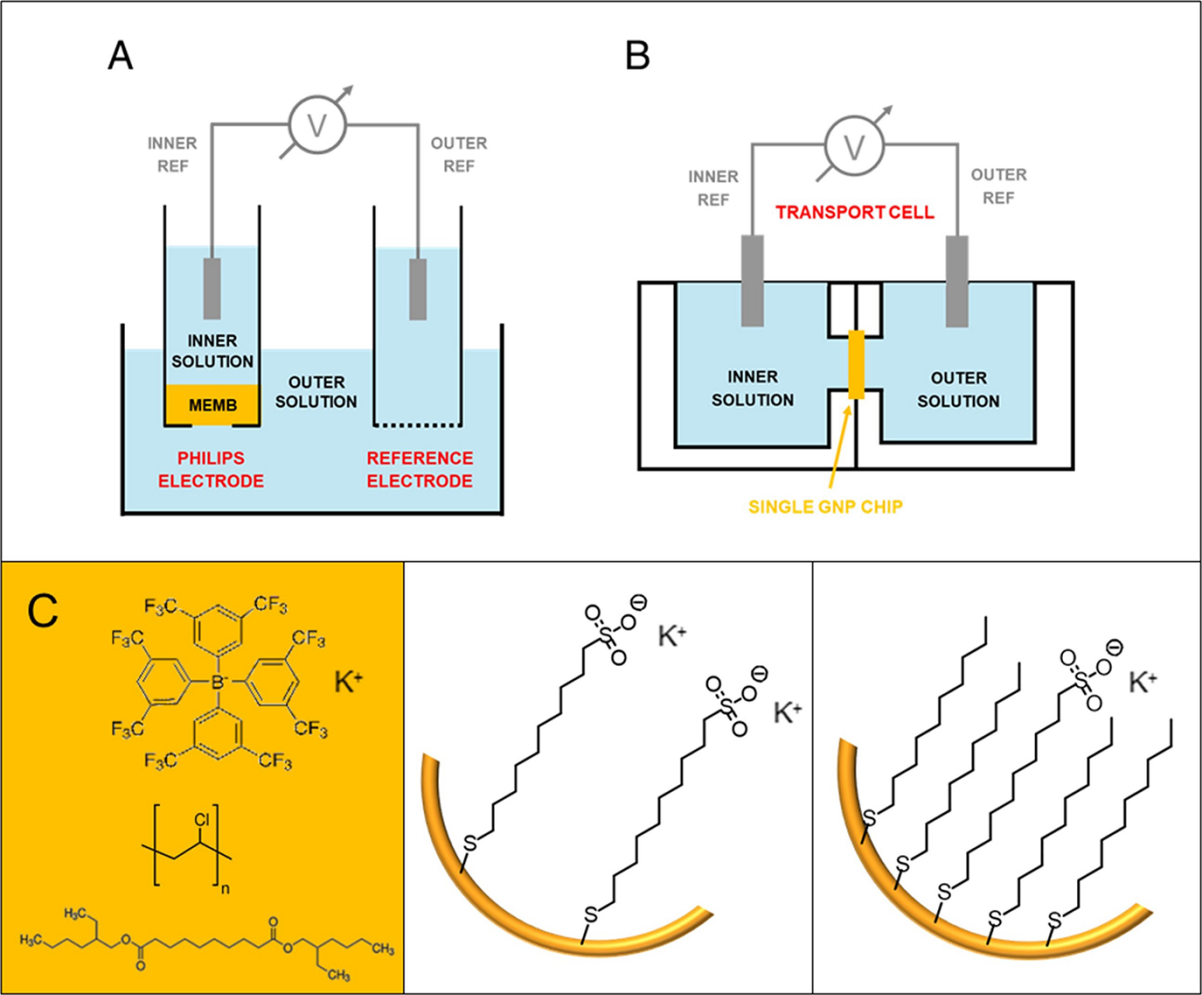
Electrochemical cell setups. (A) PVC and multipore GNP membranes (MEMB) were mounted in Philips electrode bodies filled with aqueous inner solutions and immersed together with a reference electrode in an aqueous outer solution. (B) Single GNP chips were mounted in two-chamber transport cells. The chambers of the cell were filled with aqueous solutions and electrically contacted with reference electrodes. (C) Schematic composition (from left to right) of the PVC, hydrophilic multipore GNP, and hydrophobic multipore GNP membranes.

The electrical resistance of single GNPs was measured in KCl solutions by cyclic voltammetry (CV) using a Gamry Reference 600 Potentiostat in a two-electrode setup with a scan rate of 50 mV s^–1^ between −200 and +200 mV vs the open circuit potential (OCP). The resistances were determined as the inverse of the slopes of the current–voltage curves at the OCP.

The membrane potential of the single GNPs was measured in continuously stirred outer solutions with the exponential dilution method^56,57^ using a 16-channel high-input impedance (10^15^ Ω) Lawson Laboratories voltmeter in the same transport cell setup. The membrane potential (E)–log concentration curves were transformed into membrane potential (E)–log activity curves using the two-parameter Debye–Hückel approximation.^58^

### Electrochemical Measurements of GNP and PVC Membranes

The electrical resistance of the GNP and PVC membranes was measured by electrochemical impedance spectroscopy (EIS) using a Gamry Reference 600 Potentiostat in a two-electrode setup from 100 kHz to 0.1 Hz at a DC bias of 0 mV with an AC amplitude of 30 mV. The Philips electrode bodies hosting the membrane were filled with an inner solution of 10^–2^ M KCl and, together with a double-junction Ag/AgCl/3 M KCl//1 M KCl//reference electrode, immersed in an outer solution of identical composition. The Nyquist plots of the membranes showed two incomplete semicircles, with the arc at higher frequencies indicative of the solution and reference electrode impedance and at lower frequencies of the cation-exchanger membrane. The membrane resistance was determined from the lower-frequency semicircle by fitting an equivalent circuit model using Gamry Echem Analyst (version 6.03).^50^

The membrane potentials were measured using a 16-channel high-input impedance (10^15^ Ω) Lawson Laboratories voltmeter with the same reference electrode but in varied, continuously stirred outer solutions. The membrane potential (E)–log concentration curves were transformed into membrane potential (E)–log activity curves in the same way as for the single GNPs.^58^

## Results and Discussion

To evaluate ion selectivity, the ion-exchanger membranes were mounted into Philips electrode bodies, with one side exposed to an inner solution of constant composition (10^–3^ M KCl) and the other to outer solutions of varying composition (Figure 1). This setup created a system analogous to a concentration cell across each membrane, with reference electrodes immersed in both the inner and outer solutions. By recording the electromotive force of the cell and correcting for the potential difference between the reference electrodes, we determined the equilibrium membrane potential in the various outer solutions. These measurements enabled the construction of potentiometric calibration curves for different cations, spanning from highly lipophilic (Et_4_N^+^) to strongly hydrophilic (Li^+^).

If an ion-exchange membrane is perfectly selective for ion *I*, the membrane potential is governed by the electrochemical equilibrium of *I* across the membrane. As the ion is transported across the membrane due to its activity gradient, a counteracting potential gradient develops because of the separation between the ion and its counterion in solution. The potential difference that counterbalances the activity gradient, resulting in zero net current, is the equilibrium membrane potential, *E_m_*, given as

1 $$E_{m}=\varphi_{in}-\varphi_{out}=\frac{RT}{z_{I}F}\ln\frac{a_{I,out}}{a_{I,in}}$$

where *φ* is the electric potential, *R* is the universal gas constant, *T* is the absolute temperature, *F* is the Faraday constant, *z_I_* is the charge number, and *a_I_* is the activity of ion *I*. The subscripts *in* and *out* refer to the inner and outer solution separated by the membrane. If the concentration of ion *I* is constant on one side of the membrane (e.g., in the inner solution), the equation simplifies to

$$E_{m}=E_{I}^{0}+\frac{RT}{z_{I}F}\ln a_{I,out}=E_{I}^{0}+s\log a_{I,out}$$ 2

where $E_{I}^{0}$ and $s_{I}$ are constants that represent the intercept and the slope of the membrane’s potentiometric calibration curve for the ion *I*. We fitted eq 2 to the experimental calibration curves, obtaining a pair of $E_{I}^{0}$ and *s* values for each membrane’s response to each tested ion.

As real membranes are not perfectly selective, multiple ions can influence the membrane potential simultaneously. To quantify the selectivity of the ion-exchange membranes, we applied the widely used empiric Nikolsky–Eisenman formalism:

3 $$E_{m}=E_{I}^{0}+s\log\left(a_{I,out}+K_{I,J}^{pot}a_{J,out}\right)$$

where *I* is the primary ion (K^+^ in this case) to which all other same-charge ions (*J*) are compared (*z_I_=z*_J_), and $K_{I,J}^{pot}$ is the potentiometrically determined selectivity coefficient.^54^ The $\log K_{K,J}^{pot}$ determined by the separate solution method^59^ for every membrane and interfering ion is compiled in Table 1.

**Table 1:** Potentiometrically Determined Selectivity Coefficients of Plasticized PVC and Hydrophilic and Hydrophobic 6 nm- and 20 nm-Diameter GNP Cation-Exchanger Membranes

	$$\log K_{K,J}^{pot}$$				
*J*	PVC hydrophobic	6 nm GNP hydrophilic	20 nm GNP hydrophilic	6 nm GNP hydrophobic	20 nm GNP hydrophobic
Et_4_N^+^	5.5	–1.6	–0.2	0.2	–0.8
Me_4_N^+^	3.0	–1.1	0.2	–0.3	0.0
K^+^	0.0	0.0	0.0	0.0	0.0
Na^+^	–3.0	–0.5	0.0	0.1	0.1
Li^+^	–2.7	–0.8	–0.1	0.0	–0.1
H^+^	–2.5	0.3	0.0	1.3	0.9

If the interaction between the ions and the ion exchanger is purely electrostatic, two limiting cases of selectivity among same-charge ions exist. When ions must dehydrate to enter the membrane, such as for hydrophobic bulk polymer membranes, a Hofmeister series scaling with hydration energy should arise.^54^ The extraction into the hydrophobic phase involves dehydration followed by solvation within the membrane. Given that the differences in dehydration energies are larger than the differences in solvation energies and both follow the same trend among the ions, the order of ion partition coefficients follows the order of dehydration energies.^54,60^ Accordingly, the cation-exchanger PVC membranes (Figure 2A) responded to ions in order of decreasing lipophilicity (increasing dehydration energy):

$$Et_{4}N^{+}>Me_{4}N^{+}\gg K^{+}>Na^{+}\approx H^{+}>Li^{+}$$

**Figure 2:**
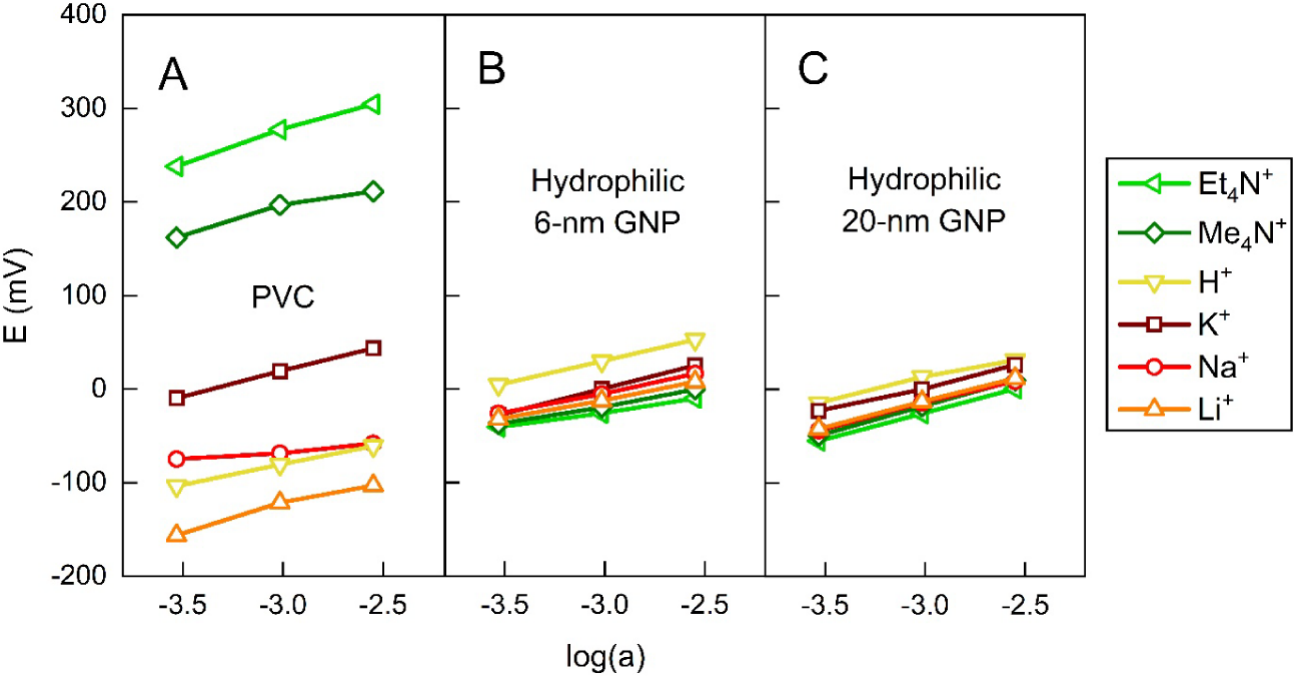
Potentiometric response of plasticized PVC (A) and hydrophilic 6 nm- (B) and 20 nm-diameter (C) GNP cation-exchanger membranes. The mean (*n* = 3) membrane potential measured in an outer solution containing the chloride salt of the respective cation is shown for several cations with varying lipophilicities.

Notably, the PVC-based membranes were 10^3^–10^5^ times more selective (as defined by $K_{K,J}^{pot}$ ) for tetraalkylammonium ions than potassium ion (Table 1). Overall, the selectivity coefficients of cation-exchanger plasticized PVC membranes ranged from ca. 10^5^ to 10^–3^ for the different monovalent ions by preserving the Hofmeister order.

In the other limiting case, no discrimination based on lipophilicity is expected when ions can permeate the membrane without dehydration. Ion-exchanger membranes comprising electrically charged hydrophilic nanopores large enough to accommodate hydrated ions (i.e., >1 nm) were shown to respond equally to same-charge ions, acting merely as charge-based filters.^40,41^ We produced such water-filled, cation-exchanger channels by decorating gold nanopore membranes (GNP membranes) with negatively charged sulfonate-bearing molecules (MDS). The water contact angle measured on MDS-modified flat gold surfaces was 15.2 ± 1.9° (*n* = 3), confirming its hydrophilicity (Figure S1C). The MDS-decorated GNP membranes acted as cation exchangers, producing positive-slope potentiometric calibration curves (Figure 2B,C). They lacked Hofmeister selectivity, as expected, giving almost equal responses to the tested cations regardless of their lipophilicity, with a slight preference for H^+^ (Table 1). This selectivity pattern is reminiscent of the behavior of Nafion membranes.^61^ In hydrated Nafion, cation permselectivity is established by the negatively charged water-filled channels, with a slight preference for H^+^ stemming from the anomalously high mobility of protons due to the Grotthuss mechanism.^62,63^ Increasing the pore diameter of the GNP membranes (from 6 to 20 nm) led to only minute changes in selectivity (Figure 2B,C, Table 1), indicating that ion sieving is absent in this size regime.

Having established that hydrophilic cation-exchanger GNP membranes do not discriminate ions based on hydration energy, we next investigated whether rendering the nanopore walls hydrophobic would induce Hofmeister selectivity. To this end, water contact angles were measured on planar gold surfaces for different cation exchanger (NaMDS) and hydrophobic thiol derivative (1-decanethiol, DT) mole ratios in the ethanolic solution used for surface modification (Figure S1A), and by using Cassie equation^64^ the fractional surface coverages of the thiol molecules on the gold surface was calculated (Figure S1B). Based on the results hydrophobic cation-exchanger nanopores were prepared by forming binary SAMs on GNP membranes using a 1:1 mixture of NaMDS and DT, which robustly provided on identically modified planar gold surfaces both a sufficiently hydrophobic surface with a water contact angle of 85.1 ± 0.5° (*n* = 3), and a 20.4% fractional surface coverage of MDS, indicating that cation-exchanger sulfonate groups remain present on the hydrophobic gold surface (the detailed calculation is presented in the SI). In comparison, surfaces coated solely with MDS and DT exhibited contact angles of 15.2 ± 1.9° and 98.0 ± 0.3°, respectively (Figure S1C,D). Consistent with the water contact angle measurements, the hydrophobic GNP membranes modified with the 1:1 NaMDS/DT mixture retained cation-permselectivity, as evidenced by their positive-slope potentiometric calibration curves (Figures 3B and S2B).

**Figure 3:**
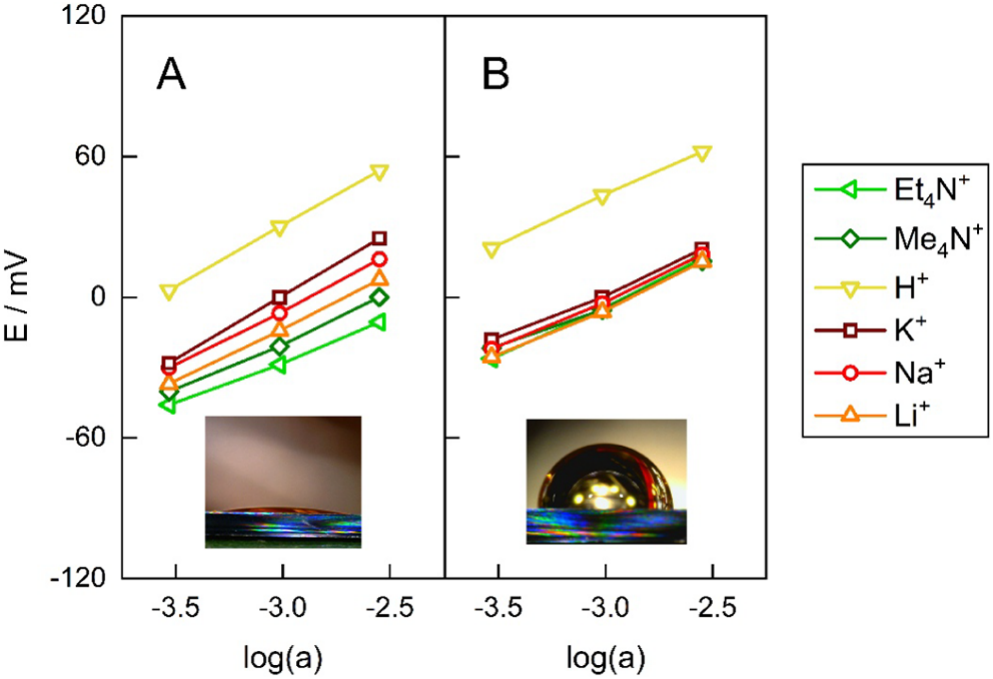
Potentiometric response of hydrophilic (A) and hydrophobic (B) 6 nm-diameter cation-exchanger GNP membranes. The mean (*n* = 3) membrane potential measured in an outer solution containing the chloride salt of the respective cation is shown for several cations with varying lipophilicities. The insets show the wettability by water of flat gold surfaces subjected to the same functionalization.

Remarkably, the hydrophobic cation-exchanger GNP membranes exhibited no Hofmeister selectivity, behaving very similarly to the hydrophilic cation-exchanger membranes (Figures 3 and S2, Table 1). The hydrophobic nanopore membranes showed no preference for lipophilic tetraalkylammonium ions, in sharp contrast to the PVC membranes (Figure 2A). The similarity to hydrophilic nanopores and the contrast with hydrophobic bulk polymer membranes is reinforced by the hydrophobic nanopores’ selectivity for divalent cations over monovalent cations (Figure S3). As divalent ions are more strongly hydrated, hydrophobic bulk polymer ion-exchanger membranes generally prefer monovalent ions.^65^ The selectivity for H^+^ was also retained and even slightly enhanced compared to the hydrophilic GNP membranes. As with the hydrophilic GNP membranes, changing the pore diameter from 6 to 20 nm did not significantly alter the selectivity (Figures 3 and S2).

The absence of Hofmeister selectivity suggests that ions remain hydrated during their transfer into the hydrophobic GNP membrane. One possible explanation is that the nanopores, despite their hydrophobic surface, are flooded by bulk aqueous electrolytes in contact with the membrane. Alternatively, the hydrophobicity may be sufficient to exclude bulk water while still permitting the entry of hydrated ions, thereby preserving the electroneutrality of the nanopore interior. To test whether bulk electrolyte penetrates the hydrophobic nanopores, we measured the electrical resistance of both hydrophilic and hydrophobic GNP membranes in 10^–2^ M KCl. Three-millimeter-diameter disks of the hydrophilic 6 nm GNP membrane exhibited a mean resistance of 59 kΩ, whereas geometrically identical hydrophobic membrane disks showed a much higher resistance of 15 MΩ, indicating a sharp change in the composition of the nanopore interior. Considering the membrane thickness (6 μm) and pore density (6 × 10^8^ pores/cm^2^), 15 MΩ corresponds to a specific pore resistance of ∼3 × 10^5^ Ω cm, equivalent to the resistance of ∼2 × 10^–5^ M aqueous KCl. This indicates that the ion concentration inside the hydrophobic nanopores is more than 2 orders of magnitude lower than in the bulk solution, suggesting that the hydrophobic pores are not flooded by the surrounding electrolyte.

The calculation above assumes that all nanopores in the GNP membrane are identical. In practice, however, the stochastic nature of the track-etching process produces a distribution of pore sizes and interpore distances (Figure S4), with a small fraction of pores even overlapping to form anomalously large channels. It is conceivable that these larger channels remain flooded in the hydrophobic GNP membranes, while the regular-sized pores exclude bulk electrolytes. Such large, water-filled channels could effectively “short-circuit” the smaller, occluded nanopores, thereby preserving the nonselectivity observed for hydrophilic GNP membranes. At the same time, the exclusion of electrolytes from the smaller pores would explain the markedly increased membrane resistance.

To test the possibility that nonidealities in the nanopore ensemble eliminate Hofmeister selectivity in multipore membranes, we evaluated the ion selectivity of single gold nanopores (single GNPs) subjected to the same surface modifications as the multipore GNP membranes—namely, NaMDS for hydrophilic cation-exchanger nanopores and a 1:1 mixture of NaMDS and DT for hydrophobic ones. The modified single GNP chips were mounted in two-chamber transport cells, with an “inner” solution of constant composition (10^–3^ M KCl) on one side and varying “outer” solutions on the other (Figure 1), in a setup analogous to that used for the multipore ion-exchanger membranes. The potentiometric calibration curves of hydrophilic and hydrophobic single GNPs (Figure 4) closely resembled those of their multipore counterparts, showing positive slopes and small curve offsets (except for H^+^). These results demonstrate that the absence of Hofmeister selectivity in hydrophobic cation-exchanger multipore GNP membranes (Figure 4B) is intrinsic to this type of pore and not an artifact arising from nanopore ensemble nonuniformities.

**Figure 4:**
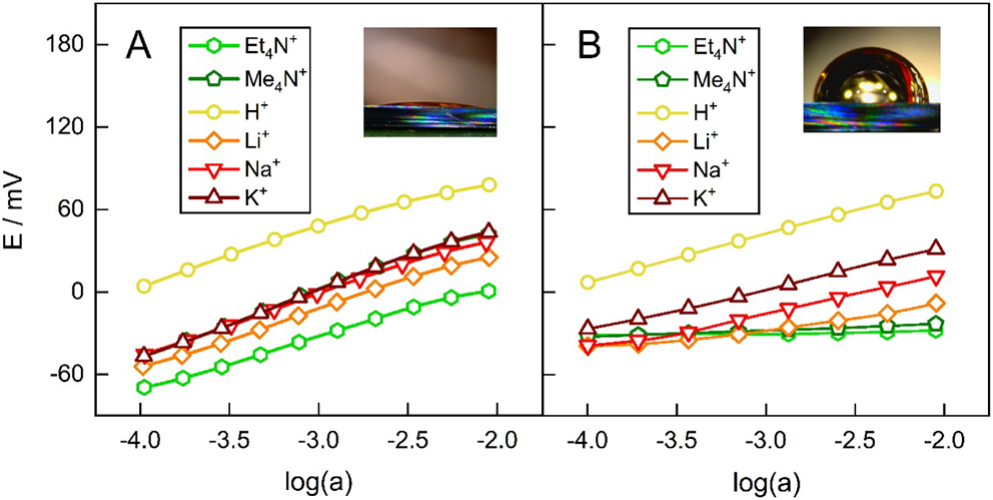
Potentiometric response of hydrophilic (A) and hydrophobic (B) 20 nm-diameter cation-exchanger single GNPs. Each curve was recorded with exponential dilution (1 symbol shown for every 100 points measured) of 10^–2^ M outer solutions containing only the chloride salt of the respective cation. The insets show the wettability by water of flat gold surfaces subjected to the same functionalization.

To investigate whether the surrounding aqueous electrolyte floods the hydrophobic single GNPs, we measured the electrical resistance of individual nanopores as a function of bulk KCl concentration (Figure 5). For hydrophilic single GNPs, the resistance remained constant at ∼3 GΩ below 10^–3^ M KCl but decreased sharply at higher concentrations. The constant resistance at low concentrations is determined by the mobile counterions that balance the nanopore’s surface charge. If bulk electrolyte enters the pore, resistance should decrease once the external KCl concentration exceeds this equivalent counterion concentration. Indeed, the linear decrease in resistance above 10^–2^ M indicates that the hydrophilic nanopores are flooded by the surrounding electrolyte. By contrast, the resistance of hydrophobic single GNPs remained constant at ∼13 GΩ across all concentrations tested. The higher constant resistance in dilute solutions reflects the reduced surface charge density of the hydrophobic nanopores, consistent with the lower slope of their potential responses (Figure 4B). Crucially, the fact that the resistance remains independent of the bulk KCl concentration confirms that the surrounding aqueous electrolyte does not flood the hydrophobic single GNPs.

**Figure 5:**
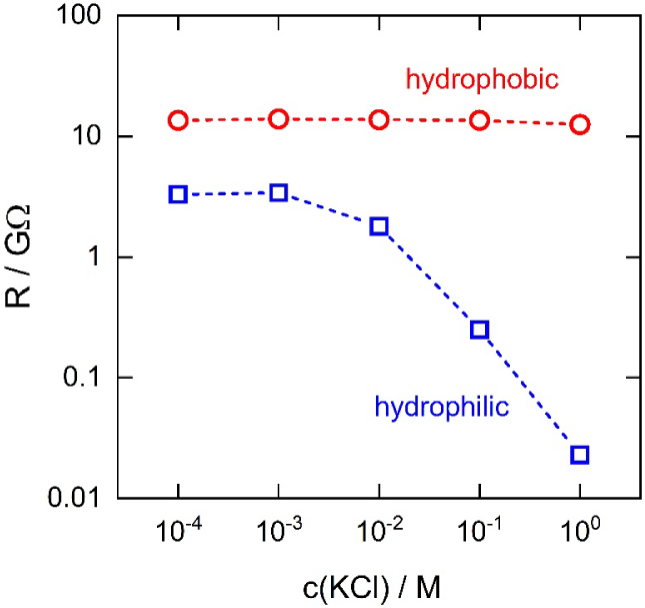
Electrical resistance of hydrophilic (blue squares) and hydrophobic (red circles) cation-exchanger 20 nm-diameter single nanopores as a function of KCl concentration in the surrounding aqueous solution. Resistances were calculated from the slope of cyclic voltammograms recorded around the open circuit potential.

Consequently, Hofmeister selectivity seems to be absent from the hydrophobic cation-exchanger GNPs despite the exclusion of bulk aqueous electrolytes. The lack of selectivity indicates that all tested ions, regardless of their lipophilicity, face similar energetic costs during their transfer into the membrane. As the ions’ solvation energies in hydrophobic phases are significantly smaller than their hydration energies, it is unlikely that the interactions between the ions and the hydrophobic SAM could compensate for the differences in dehydration energy. This suggests that the ions infiltrate the nanopore without dehydration, indicating that the hydrophobic SAM cannot prevent the entry of hydrated ions that are attracted by the nanopore’s surface charge. Numerous studies have found that hydrophobic nanoconfinements can contain water in various forms^66–69^ with properties that sharply differ from those of bulk water.^70^ The preservation or slight enhancement of H^+^ selectivity in the hydrophobic GNPs is also consistent with the presence of confined water in the hydrophobic nanopore lumen.^71^ In this scenario, the role of hydrophobicity in the synthetic ion channels^50,51^ might be not to dehydrate ions but to reduce their concentration in the pore lumen by excluding *bulk* electrolytes, establishing the molar excess of the surface-bound ionophore. A molar excess of ionophore relative to the amount of mobile counterions is a requisite of ionophore-dominated selectivity in ion-selective membranes.^65^

Regardless of its underlying cause, the absence of inherent Hofmeister selectivity could offer significant advantages by liberating ion-selective nanopores from interference by lipophilic species—a pervasive limitation in conventional hydrophobic, bulk polymer-based ion-selective membranes. As observed previously,^50,51^ this enables synthetic ion channels to discriminate lipophilic interferents as effectively as hydrophilic ones, thereby broadening the range of sample types amenable to ion-selective potentiometry. Furthermore, in the absence of a direct contribution from surface hydrophobicity, ion selectivity in these systems should be governed almost entirely by the ionophore. This decoupling of selectivity from nonspecific lipophilic interactions offers a pathway toward the rational design of functionalized nanopore-based ion-selective sensors.

## Conclusions

In this study, we systematically investigated the role of hydrophobicity in the ion selectivity of nanometer-wide ion-exchanger nanopores. By comparing the potentiometric responses of structurally identical hydrophilic and hydrophobic sulfonated gold nanopores—whose diameters are substantially larger than those of hydrated ions—we found that neither exhibited Hofmeister selectivity. This behavior of hydrophobic cation-exchanger GNPs contrasts sharply with that of conventional hydrophobic bulk polymer membranes and subnanometer pores. The absence of Hofmeister selectivity in both multipore membranes and single nanopores demonstrates that surface hydrophobicity alone does not induce ion dehydration in nanometer-wide pores. Importantly, the lack of discrimination against lipophilic ions may be advantageous in ligand-modified nanopore-based ion sensing, as it eliminates the common interference from lipophilic species that affects conventional polymeric ion-selective membranes, ensuring that ion selectivity is governed solely by the ligand. Moreover, hydrophobic cation-exchanger nanopores could find applications as detectors in ion separation and in the determination of cumulative or total ion concentrations in biological fluids, measurements that would otherwise be biased by minute amounts of lipophilic ions when conventional hydrophobic polymer ion-exchanger membranes are used.

## Supplementary Material



## Notes

Water contact angle measurements and fractional surface coverage calculations, monovalent over monovalent ion selectivity of hydrophilic and hydrophobic 20 nm-diameter cation-exchanger GNP membranes, divalent over monovalent ion selectivity of hydrophilic and hydrophobic 6 nm-diameter GNP cation-exchanger membranes, SEM image of a representative track-etched polycarbonate filter membrane, detailed description of GNP membrane fabrication, characterization and electrochemical measurements, detailed description of single GNP fabrication, characterization and electrochemical measurements (PDF)
