# Supplementary material for: Absence of Hofmeister Selectivity in Hydrophobic Ion-Exchanger Nanopores
Source: Anal Chem. 2025 Dec 1;97(49):27289–97. doi: 10.1021/acs.analchem.5c05239 (PMC12713612; doi:10.1021/acs.analchem.5c05239)
Supplement: Supplementary file 1 [file ac5c05239_si_001.pdf]

Supporting Information

Absence of Hofmeister Selectivity in Hydrophobic Ion-Exchanger Nanopores

Gergely T. Solymosi[1], Tünde Kis[1], Péter Fürjes,[2] Róbert E. Gyurcsányi[1,3]*

[1]BME Lendület Chemical Nanosensors Research Group, Department of Inorganic and Analytical Chemistry, Budapest University of Technology and Economics, Műegyetem rkp. 3, H-1111 Budapest, Hungary

[2]Institute of Technical Physics and Materials Science, HUN-REN Centre for Energy Research, Konkoly Thege Miklós út 29-33, H-1121 Budapest, Hungary

[3]HUN-REN-BME Computation Driven Chemistry Research Group, Department of Inorganic and Analytical Chemistry, Budapest University of Technology and Economics, Műegyetem rkp. 3, Budapest 1111, Hungary

*gyurcsanyi.robert@vbk.bme.hu

Table of contents

1. Water contact angle measurements and fractional surface coverage calculations
2. Monovalent-over-monovalent ion selectivity of hydrophilic and hydrophobic 20-nm-diameter cation-exchanger GNP membranes
3. Divalent-over-monovalent ion selectivity of hydrophilic and hydrophobic 6-nm-diameter GNP cation-exchanger membranes
4. SEM image of a representative track-etched polycarbonate filter membrane
5. Detailed description of multipore GNP membrane fabrication, characterization and electrochemical measurements
6. Detailed description of single GNP fabrication, characterization and electrochemical measurements

1. Water contact angle measurements and fractional surface coverage calculations

To find the NaMDS:DT ratio in the modifying solution that results in hydrophobic surfaces while preserving sufficient charge density, water contact angles (WCAs) were measured on gold surfaces modified with different molar ratios of NaMDS and DT (Figure S1A).

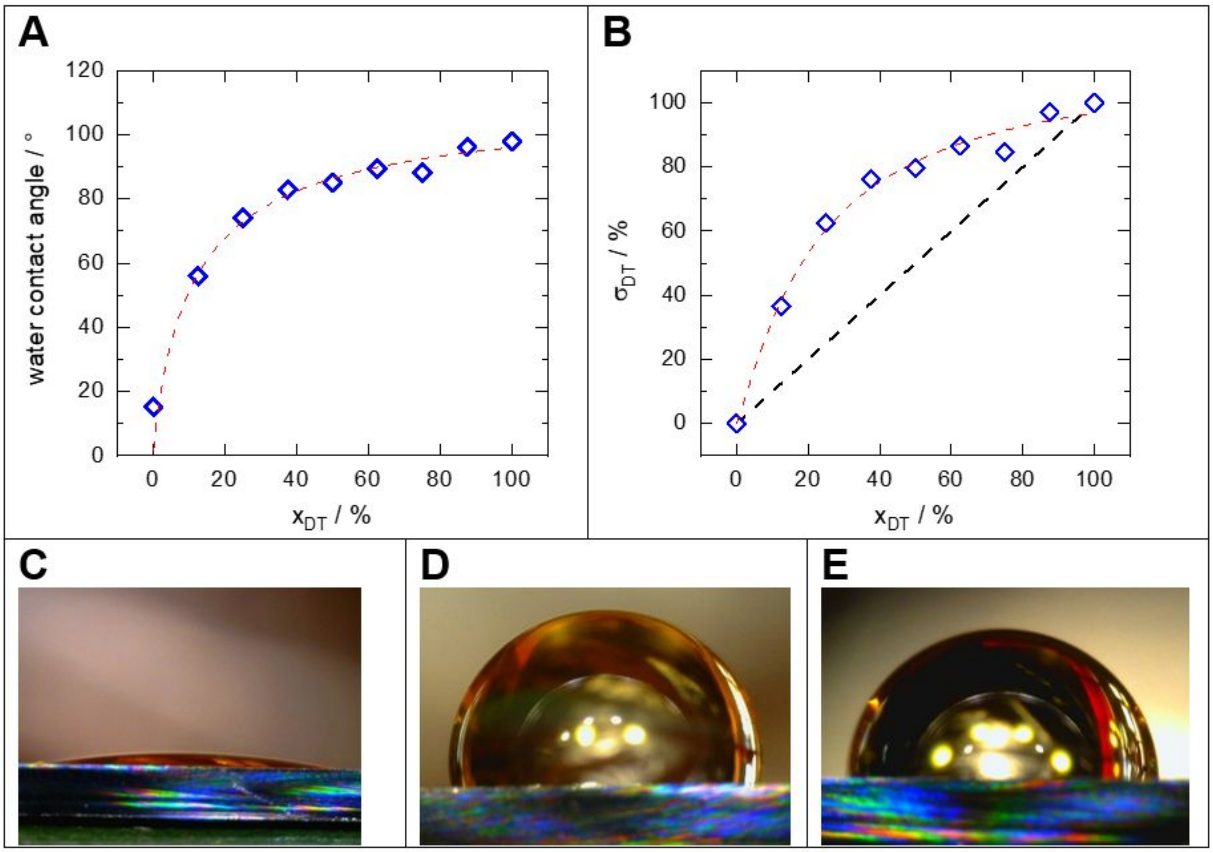

Figure S1. Water contact angles measured on flat gold surfaces modified with 0.1 mM (total concentration) ethanolic solution of binary mixtures of NaMDS and DT at different molar ratios. The dependence of (**A**) WCA and (**B**) the fractional surface coverage of DT (σ_{DT}) estimated using the Cassie equation on the mole fraction of DT (x_{DT} (%)) in the modifying solution. Water droplets on gold surfaces modified with NaMDS (**C**), DT (**D**), and 1:1 molar ratio mixture of NaMDS and DT (**E**). The respective contact angles were determined to be 15°, 98°, and 85°.

From the WCA–x_{DT} curve, the dependence of the fractional surface coverage of DT (σ_{DT}) on its mole fraction in the modifying solution (x_{DT}) (Fig. S1 B) could be calculated using the Cassie equation:

$$\cos\theta_{mix} = \sigma_{MDS}\cos\theta_{MDS} + \sigma_{DT}\cos\theta_{DT}$$

Fig. S1B clearly shows the preferential incorporation of the hydrophobic linear DT into the binary SAM, confirming a marked deviation from its mole fractions *in the modifying solution*.

For the case of 1:1 mole ratio of NaMDS and DT in the modifying solution, based on the determined WCA (85°), and the WCAs of the surfaces modified solely with NaMDS (15°) and DT (98°), the Cassie equation yielded an MDS fractional surface coverage of ~20%, and ~80% for DT:

$$\sigma_{DT} = (1 - \sigma_{MDS})$$

$$\cos(85°) = \sigma_{MDS}\cos(15°) + (1 - \sigma_{MDS})\cos(98°)$$

$$\sigma_{MDS} = \frac{\cos(85°) - \cos(98°)}{\cos(15°) - \cos(98°)} = 0.204$$

$$\sigma_{DT} = (1 - \sigma_{MDS}) = 0.796$$

2. Monovalent-over-monovalent ion selectivity of hydrophilic and hydrophobic 20-nm-diameter cation-exchanger GNP membranes

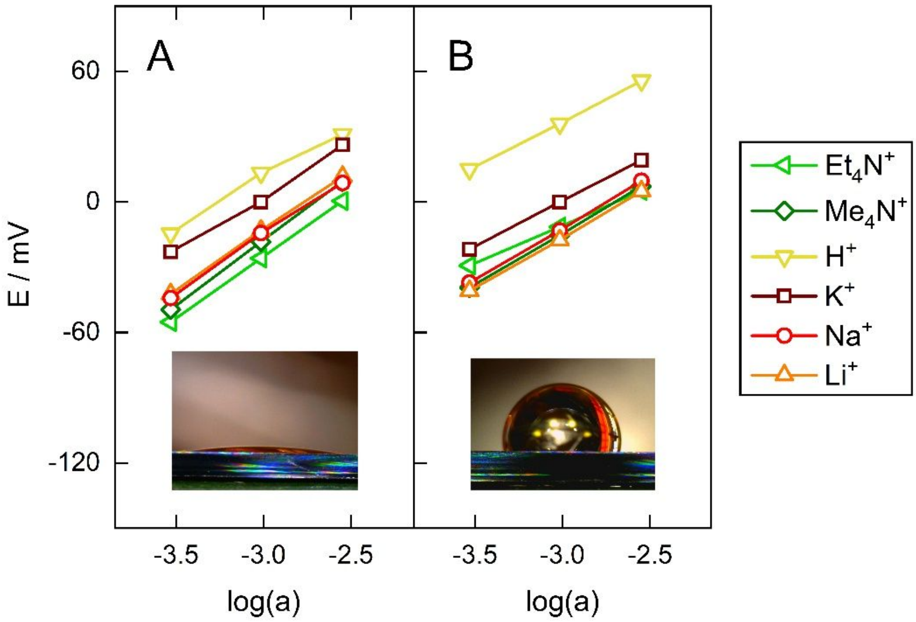

Figure S2. Potentiometric response of hydrophilic (**A**) and hydrophobic (**B**) 20-nm-diameter cation-exchanger GNP membranes. The hydrophilic GNP membranes were functionalized with MDS. The hydrophobic GNP membranes were modified with a 1:9 mixture of NaMDS and DT. The mean (n=3) membrane potential measured in an outer solution containing the chloride salt of the respective cation

is shown for several cations with varying lipophilicities. The insets show the wettability by water of flat gold surfaces subjected to the same functionalization.

3. Divalent-over-monovalent ion selectivity of hydrophilic and hydrophobic 6-nm-diameter GNP cation-exchanger membranes

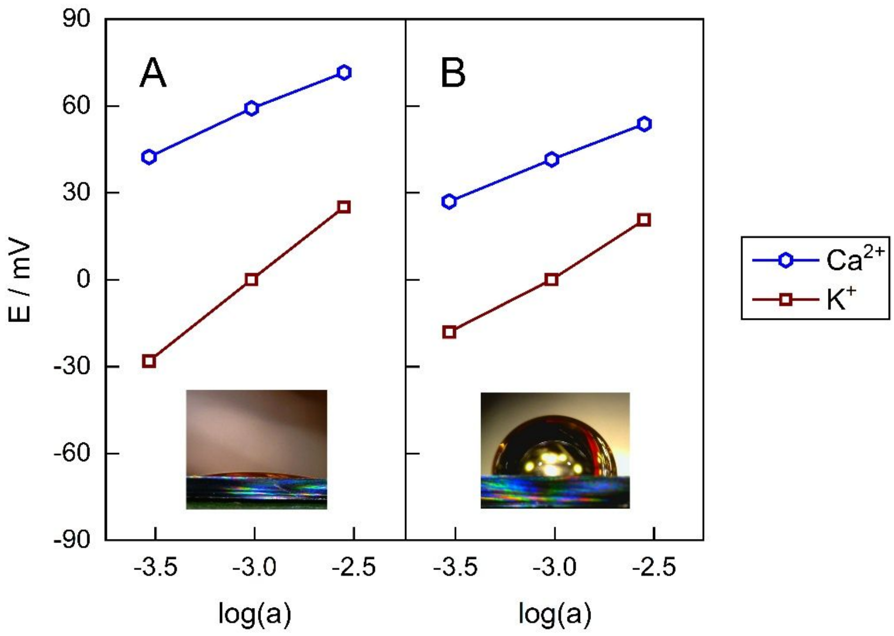

Figure S3. Divalent-over-monovalent ion selectivity of hydrophilic (**A**) and hydrophobic (**B**) 6-nm-diameter GNP cation-exchanger membranes. The mean (n=3) membrane potential measured in outer solutions containing $CaCl_2$ or KCl are shown. The insets show the wettability by water of flat gold surfaces subjected to the same functionalization.

4. SEM image of a representative track-etched polycarbonate filter membrane

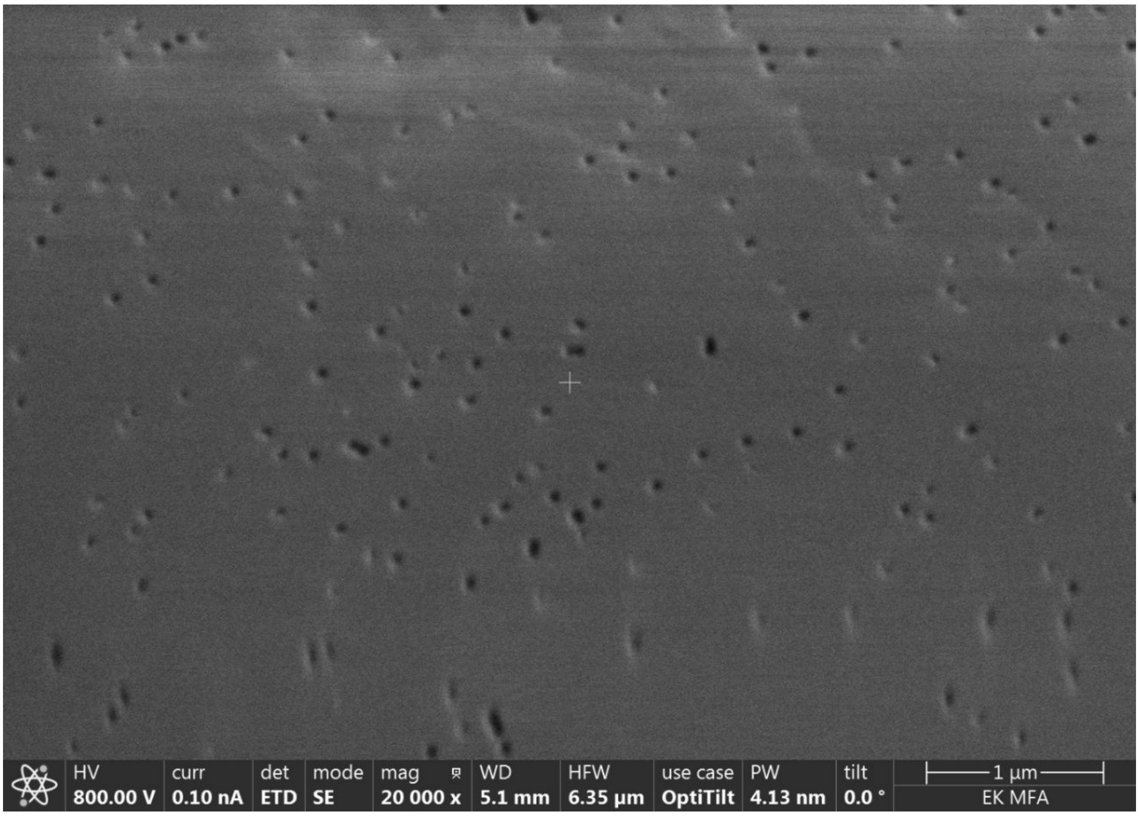

Figure S4. SEM image of a representative track-etched polycarbonate filter membrane used as the substrate for fabricating GNP membranes by electroless gold plating.

5. Detailed multipore GNP membrane fabrication, characterization and electrochemical measurements

Fabrication of GNP membranes. Multipore GNP membranes were made via electroless gold plating of track-etched filter membranes. Hydrophilic (polyvinylpyrrolidone-coated) polycarbonate filter membrane disks with a diameter of 25 mm, thickness of 6 µm, pore density of $6 \cdot 10^8$ pore/cm^2, and nominal pore diameter of 30 nm were obtained from Cytiva (US). We used a modified version[1] of the electroless gold plating protocol developed by Martin's group.[2] First, the filter membranes were soaked in methanol for 5 minutes, then immersed for 45 minutes in a 1:1 methanol–water mixture containing 0.026 M $SnCl_2$ and 0.07 M trifluoroacetic acid. During this step, the Sn^{2+} ions adsorb to the membrane surface via complexation with the functional groups of the polyvinylpyrrolidone layer. Subsequently, the membranes were rinsed with fresh methanol thrice for 2 minutes. In the second step, the membranes were placed for 5 minutes in an aqueous solution containing 0.029 M $[Ag(NH_3)_2]^+$, then washed in methanol two

times for 2 minutes and dried in a N_2 atmosphere for 15 minutes. The Sn^{2+} ions adsorbed to the surface reduce Ag^+ ions, forming nanoscopic Ag^0 particles on the surface and sensitizing the membrane for gold deposition. In the final step, the sensitized membranes were immersed in the electroless gold plating solution at 1 °C for 180–360 minutes. The plating time affects the amount of gold deposited, thus providing a convenient way to adjust the pore size. The plating solution contained 7.9 mM $Na_3Au(SO_3)_2$ (2.50 ml Oromerse SO Part B / 100 ml plating solution), 0.025 M $NaHCO_3$, 0.127 M Na_2SO_3, and 0.625 M formaldehyde. The plating solution's pH, initially around 12.0, was titrated to 10.0 with 1 M sulfuric acid. Finally, the gold-plated membranes were rinsed with deionized water for 5 min and dried in an ambient atmosphere.

Determination of GNP membrane pore size. The average inner diameter of the nanopores in the GNP membranes was determined by N_2 gas permeation measurements.[3] The GNP membrane was mounted into a filter membrane holder (Whatman, UK) and exposed to a pressurized stream of nitrogen gas. By measuring the flux of N_2 across the membrane, the average inner diameter of the nanopores could be calculated based on the Knudsen equation:

$$d_p = \left\{ 6Q\left(\frac{MRT}{2\pi}\right)^{\frac{1}{2}} \frac{l_p}{N\Delta p} \right\}^{\frac{1}{3}}$$

where d_p is the average inner diameter of the nanopores, Q is the flux of N_2 through the membrane, N is the number of pores, M is the molecular weight of N_2, R is the universal gas constant, T is the absolute temperature, l_p is the thickness of the membrane (i.e., the approximate length of the pore), and Δp is the pressure drop across the membrane.

GNP membrane electrochemical cell assembly. 7-mm-diameter disks of the functionalized GNP membranes were mounted in Philips electrode bodies containing a Ag/AgCl wire as the internal reference electrode and aqueous KCl as internal solution. To ensure proper sealing, the GNP membranes were sandwiched between two silicon rubber O-rings. The O-ring on the external side had an outer diameter of 7 mm and an inner diameter of 3 mm. The O-ring facing the internal solution in the Philips electrode had an outer and inner diameter of 6 and 3 mm, respectively.

GNP membrane resistance measurement. The electrical resistance of the GNP membranes was measured by electrochemical impedance spectroscopy (EIS) using a Gamry Reference 600 Potentiostat in a two-electrode setup. The Philips electrode hosting the membrane was filled

with an inner solution of 10^{-2} M KCl and, together with a double-junction Ag/AgCl/3M KCl//1M KCl//, immersed in an outer solution of identical composition. The Philips electrode was attached to the joint working and working sense leads of the potentiostat, while the reference electrode was connected to the joint reference, counter, and counter sense leads. The impedance spectrum of the cell was recorded from 100 kHz to 0.1 Hz at a DC bias of 0 mV with an AC amplitude of 30 mV. The Nyquist plots of the membranes showed two incomplete semicircles. The arch at higher frequencies corresponds to the impedance of the solutions and reference electrodes (i.e., the cell's impedance without the membrane). The arch at lower frequencies captures the impedance of the membrane. The membrane resistance was extracted from this second semicircle by fitting an equivalent circuit model to the recorded spectra using Gamry Echem Analyst (version 6.03), as had been done before.[4]

GNP membrane potential measurement. The membrane potential was measured under zero current conditions using a 16-channel high-input impedance (10^{15} Ω) Lawson Labs potentiometer. The Philips electrode holding the GNP membrane was filled with 10^{-3} M KCl as an inner solution, immersed in a continuously stirred outer solution, and connected to the potentiometer as an indicator electrode. Its electrode potential was measured against a double-junction Ag/AgCl/3M KCl//1M KCl// reference electrode immersed in the same solution and connected to the potentiometer as a reference electrode. The membrane potential (E) was calculated from the recorded electromotive force (EMF) by subtracting the EMF measured in an outer solution that was identical to the inner solution (i.e., 10^{-3} M KCl).

6. Detailed single GNP fabrication, characterization and electrochemical measurement

Fabrication of single GNPs. The single GNPs were fabricated by focused ion beam (FIB) milling in a submicron-thick multilayer membrane[5] — consisting of a 200-nm-thick non-stoichiometric silicon nitride (SiN_x) supporting layer, a 5-nm-thick titanium (Ti) adhesion layer, and a 150-nm-thick gold (Au) layer — that was suspended on a 380-µm-thick silicon (Si) frame (Figure 7A). The membrane containing the single GNP and the Si frame are collectively referred to as the single GNP chip.

First, 200-nm-thick SiN_x layers were deposited by a Low-Pressure Chemical Vapor Deposition system (Tempress Omega M, The Netherlands) on both sides of a 3-inch-wide, 380 µm thick, <100> oriented, and double-side-polished Si substrate. Dichlorosilane and ammonia (Messer, Hungary) were used as precursors with gas flow rates of 160 sccm and 20 sccm, respectively, at 830 ºC temperature and 180 mTorr backpressure (adjusted by nitrogen injection). Then, a 5-nm-thick layer of Ti was deposited on the "inner" side (see Figure 7) as an intermediate layer for adequate adhesion, followed by a 150-nm-thick Au layer. The metal layers were deposited by electron beam evaporation in an AJA ATC Orion system (USA) at 10^{-6} mTorr chamber vacuum. Subsequently, the Au layer was patterned into a 350-µm-diameter disk by lift-off lithography, using spin-coated (at 3000 rpm) Dow Megaposit SPR 220-4.5 (United States) photoresist and a MA6 mask aligner (SÜSS MicroTec, Germany).

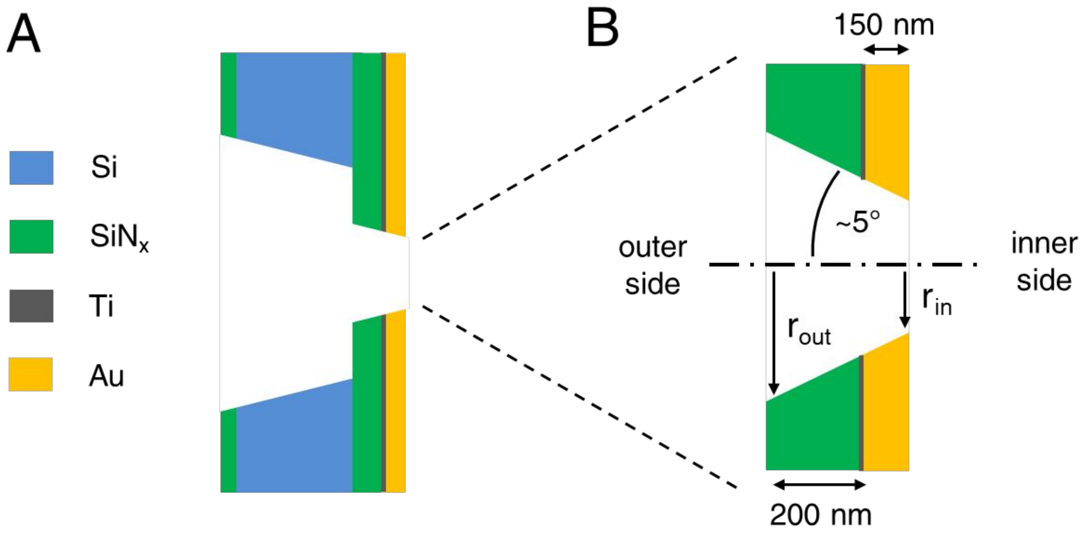

Figure S5. Schematic structure of the single GNP chip (**A**) and geometry of the single GNP (**B**). The "inner" and "outer" sides of the single GNP face the inner and outer solution, respectively, during electrochemical measurements (see Figure 1).

To expose the submicron multilayer membrane, the thick Si substrate was anisotropically etched away from the "outer" side with a ca. 50 μm/min etching rate in 23.8 m/m% KOH solution at 72 ºC. The etching window on the "outer side" was 700 × 700 μm^2, which resulted in a ca. 160 × 160 μm^2 final membrane area on the "inner" side, given the 54.7º side angle of the etched narrowing grove defined by the (100) crystal plane of the Si lattice. The etching window was exposed by removing the "outer" side SiN_x layer using CF_4/O_2 Plasma Etching in an Anelva Reactive Ion Etching equipment (Japan) with 50 mTorr chamber vacuum, 100 sccm CF_4 and 17 sccm O_2 flow rates.

To ensure the adequate conductivity of the membrane during the FIB milling, a ca. 200-nm-thick sacrificial aluminum (Al) layer was deposited on the "outer" side by electron beam evaporation at 10^{-6} mTorr chamber vacuum in an AJA ATC Orion system (USA). After singularization into 5-mm square-shaped chips by a Disco 560 (Japan) wafer dicer, the resulting single GNP chips were fixed on an electrically conducting sample holder. The Al layer was grounded to prevent the electrical charging of the membrane during the FIB milling, which could have deteriorated the pore geometry. The nanopores were drilled from the SiN_x ("outer") side of the membrane to ensure the smallest possible pore size on the Au ("inner") side using a Thermo Scientific Scios 2 DualBeam ultra-high-resolution analytical focused ion beam scanning electron microscopy (FIB-SEM) nanoprocessing system with 10^{-7} mBar chamber vacuum, 30 keV ion energy, 1.5 pA beam current, 12 s milling time, 18 pQ milling ion dose, resulting in ca. 10 nm pore radius. The FIB milling produced truncated cone-shaped nanopores with a half cone angle of 4.9±0.8° (n=4) and the wider orifice located on the "outer" (SiN_x) side of the membrane, as determined by cross-sectional TEM analysis. After the FIB milling, the sacrificial Al layer was removed at room temperature by wet chemical etching in 5% v/v phosphoric acid solution.

Determination of single GNP pore geometry. To obtain the geometry of the single GNP (Figure 7B), cross-sectional analysis was performed by high-resolution transmission electron microscopy (HRTEM/XTEM) in JEOL JEM-3010 HRTEM / XTEM (Japan)[5]. For this purpose, a single GNP array was fabricated in an identical multilayer SiNx/Ti/Au membrane structure by FIB milling in a Zeiss LEO 1540XB Cross-Beam Field Emission Scanning Electron Microscope and FIB/EBAD/IBAD nanoprocessing system using 10^{-7} mBar chamber vacuum, 30 keV ion energy, 5 pA beam current, 4 s milling time, 20 pQ milling ion dose, resulting in a ca. 20-nm pore radius. The pores in the array were filled with platinum (Pt) by Electron Beam Assisted Deposition in the Gas Injection System (GIS) with 10^{-5} mbar chamber vacuum, 5 keV

acceleration voltage, methylcyclopentadienyl-(trimethyl)platinum precursor gas, and 72 ºC source temperature to aid proper TEM lamella preparation and ensure adequate mechanical stability. The section of the multilayer membrane containing the single GNP array was etched with a focused Ga^+ ion beam using a 10^{-7} mBar chamber vacuum, 5 nA beam current for coarse milling, and 10–100 pA beam current for fine polishing, and then transferred to the TEM sample holder. The glued and fixed membrane was thinned down to a TEM lamella with a thickness of 40–100 nm by computer-controlled FIB feature milling, and XTEM imaging of the single GNP array cross-section was performed with 10^{-7} mBar chamber vacuum and 300 keV acceleration voltage.

Determination of single GNP pore diameter. The "inner" diameter (i.e., the diameter of the pore on the Au side of the membrane) was determined individually for each single GNP by scanning electron microscopy (SEM) immediately after fabrication using the same cross-beam setup of Thermo Scientific Scios 2 DualBeam FIB-SEM. A specially structured metal holder was fitted under the single GNP, and the map of secondary electron yield generated by electrons translocating the single pore was recorded. The pore radius was estimated from the secondary electron yield map by intensity profile analysis using ImageJ (National Institutes of Health, USA).

Single GNP electrochemical cell assembly. Single GNP chips were sandwiched between two disk-shaped holders that contained outward-opening cone-shaped holes in their center to allow contact between the single GNPs and the electrolyte solutions. The holders were padded with Parafilm (Bemis, US) O-rings to ensure watertight sealing between the holders and the single GNP chip. The holder-chip assembly was mounted in a two-chamber Teflon transport cell (Figure 1B). The chambers were filled with aqueous electrolyte solutions, and double-junction Ag/AgCl/3M KCl//1M KCl// reference electrodes (Metrohm, Switzerland) were immersed into the solutions for electrical contact.

Single GNP resistance measurement. The reference electrodes were connected to a Gamry Reference 600 Potentiostat in a two-electrode setup. The electrical resistances of the single GNPs were measured by cyclic voltammetry with a scan rate of 50 mV s^{-1} between −200 and +200 mV vs. the open circuit potential of the cell. The resistances were determined as the inverse of the slopes of the linear current–voltage curves.

Single GNP potential measurement. The reference electrodes were connected to a 16-channel high-input impedance (10^{15} Ω) Lawson Labs (US) potentiometer. 10^{-3} M KNO_3 was used as

the inner solution, and $5 \cdot 10^{-6}$ M KCl as the background electrolyte in the outer solution (Figure 1B). Potentiometric calibration curves were recorded in continuously stirred solutions using the exponential dilution method.[6,7] A 10^{-2} M aqueous solution of the respective cation's chloride salt was introduced into the outer chamber, then, the background electrolyte (i.e., $5 \cdot 10^{-6}$ M KCl) was pumped into the continuously stirred outer chamber using a peristaltic pump at a flow rate (w) of 5 mL/min until the measured EMF became constant (i.e., the lower detection limit was reached due to the dilution). The volume of the outer solution (V) was kept constant at 15 mL by continuously draining the outer chamber at the corresponding fluid level. Under such circumstances, the concentration (c) of the solute in the outer solution decays exponentially with time (t) from its initial value (c_0):

$$c(t) = c_0 e^{-\frac{w}{V}t} \qquad (S1)$$

If the inner solution is constant, the membrane potential depends logarithmically on the outer solution's concentration (Equation 2 in main text). Thus, in the Nernstian range of the calibration curve, the measured membrane potential changes linearly with time during the exponential dilution:

$$E(t) \approx E_I^0 + s_I \, log\left(c_0 e^{-\frac{w}{V}t}\right) = E_I^0 + s_I \, log\, c_0 - s_I \frac{w}{V} \frac{1}{ln\, 10} t = E_{I,t=0} - s_I \frac{t}{t_{0.1}} \qquad (S2)$$

with $t_{0.1}$ being the time required for the tenfold dilution of the outer solution:

$$t_{0.1} = \frac{V}{w} ln\, 10 = \frac{15\, mL}{5\, mL/min} \cdot 2.3 \approx 7\, min \qquad (S3)$$

Therefore, the recorded EMF–time curves were transformed into EMF–log concentration curves using the conversion:

$$log\, c(t) = log\, c_0 - \frac{t}{t_{0.1}} = log\, c_0 - \frac{w}{V} \frac{1}{ln\, 10} t \qquad (S4)$$

Then, OriginPro2018 was used to apply a 200-point (ca. 1-Hz) FFT filter to the raw data to remove high-frequency noise. The EMF values were subsequently shifted to obtain membrane potential (E) values by subtracting the EMF measured in an outer solution identical to the inner solution (i.e., 10^{-3} M KCl). Finally, the membrane potential (E)–log concentration curves were transformed into membrane potential (E)–log activity curves using the two-parameter Debye–Hückel approximation.[8]

References

(1) Jágerszki, G.; Gyurcsányi, R. E.; Höfler, L.; Pretsch, E. Hybridization-Modulated Ion Fluxes through Peptide-Nucleic-Acid- Functionalized Gold Nanotubes. A New Approach to Quantitative Label-Free DNA Analysis *Nano Lett.* **2007**, *7*, 1609-1612, DOI: 10.1021/nl0705438.

(2) Nishizawa, M.; Menon, V. P.; Martin, C. R. Metal Nanotubule Membranes with Electrochemically Switchable Ion-Transport Selectivity *Science* **1995**, *268*, 700-702, DOI: doi:10.1126/science.268.5211.700.

(3) Papp, S.; Jágerszki, G.; Gyurcsányi, R. E. Ion-Selective Electrodes Based on Hydrophilic Ionophore-Modified Nanopores *Angew. Chem. Int. Ed.* **2018**, *57*, 4752 - 4755, DOI: 10.1002/anie.201800954.

(4) Jagerszki, G.; Takacs, A.; Bitter, I.; Gyurcsanyi, R. E. Solid-state ion channels for potentiometric sensing *Angew Chem Int Ed Engl* **2011**, *50*, 1656-1659, DOI: 10.1002/anie.201003849.

(5) Fürjes, P. Controlled Focused Ion Beam Milling of Composite Solid State Nanopore Arrays for Molecule Sensing *Micromachines* **2019**, *10*, 774.

(6) Horvai, G.; Tóth, K.; Pungor, E. A simple continuous method for calibration and measurement with ion-selective electrodes *Anal. Chim. Acta* **1976**, *82*, 45-54, DOI: 10.1016/s0003-2670(01)82202-9.

(7) Lindner, E.; Gyurcsányi, R. E.; Buck, R. P. Tailored transport through ion-selective membranes for improved detection limits and selectivity coefficients *Electroanal.* **1999**, *11*, 695 - 702, DOI: 10.1002/(sici)1521-4109(199907)11:10/11<695::aid-elan695>3.0.co;2-g.

(8) Meier, P. C. Two-parameter debye-hückel approximation for the evaluation of mean activity coefficients of 109 electrolytes *Anal. Chim. Acta* **1982**, *136*, 363-368, DOI: 10.1016/s0003-2670(01)95397-8.
